# JC Polyomavirus in Prostate Cancer—Friend or Foe?

**DOI:** 10.3390/cancers17101725

**Published:** 2025-05-21

**Authors:** Jacek Kiś, Dominika Sikora, Mirosław J. Jarosz, Małgorzata Polz-Dacewicz

**Affiliations:** 1 Department of General and Oncological Urology, 1st Clinical Military Hospital with Outpatient Clinic in Lublin, 20-049 Lublin, Poland; jacekkis@gmail.com; 2 Department of Virology with Viral Diagnostics Laboratory, Medical University of Lublin, 20-093 Lublin, Poland; dominika.sikora@umlub.pl; 3 Faculty of Human Sciences, University of Economics and Innovation, 20-209 Lublin, Poland; miroslaw.jarosz@wsei.pl

**Keywords:** prostate cancer, JCV, EBV/JCV co-infection

## Abstract

**Background/Objectives**: Recently, many researchers have evaluated various viruses, including polyomaviruses (JCV, BKV) and EBV, as potential factors playing a role in the development and/or progression of prostate cancer (PCa), one of the most common cancers in men. Therefore, we aimed to assess the frequency of the JCPyV DNA in tissue collected from PCa patients. **Methods**: We detected the presence of viral DNA (PCR) in 49.6% of clinical samples, including 71.9% with single EBV infection and 28.1% with EBV/JCV co-infection. We did not detect BKV or a single JCV infection. Therefore, we compared patients with EBV mono-infection with EBV/JCV co-infected patients in the context of risk group, Gleason score, and TNM classification. **Results**: Our results showed differences in clinicopathological features between single EBV infection and EBV/JCV co-infection. In the group of patients with single EBV infection, most patients were classified as medium/high risk, while in the group with EBV/JCV co-infection, most patients were classified as low risk. **Conclusions**: Among patients with single EBV infection, a more advanced stage of cancer was observed than in EBV/JCV co-infection. Moreover, the level of anti-EBVCA and anti-EBNA antibodies as well as EBV load was higher in the case of single infection compared to EBV/JCV co-infection. Higher antibody levels were detected in more advanced tumor stages in single EBV infection. Does JCV only “reside” in prostate cells or is it a co-factor in EBV infection? In light of these studies, there is a need to clarify the role of JCV virus in the development and/or progression of prostate cancer.

## Simple Summary

Various viruses, including polyomaviruses (JCV, BKV) and EBV, are considered as potential factors in the development and/or progression of prostate cancer (PCa), one of the most common cancers in men. Therefore, the aim of the presented study was to assess the frequency of JCPyV DNA in PCa tissue. We detected viral DNA in 49.6% of clinical samples, including 71.9%—single EBV infection and 28.1%—EBV/JCV co-infection. We did not detect BKV or a single JCV infection. In the EBV single infection group, most patients were classified as intermediate/high risk; a higher level of anti-EBV antibodies and EBV load were found compared to EBV/JCV co-infection and a more advanced clinical stage. Does JCV only “reside” in prostate cells or is it a co-factor in EBV infection? In light of these studies, there is a need to clarify the role of JCV virus in the development and/or progression of prostate cancer.

## 1. Introduction

High rates of morbidity and mortality due to cancer set new research directions to meet the challenges of modern and effective diagnostics, therapy, and prevention of these diseases.

Prostate cancer is one of the most common cancers among men worldwide. According to IARC data, in 2022, 1,467,854 new cases of prostate cancer and 397,430 deaths from this cause were registered [1]. Also, in Poland, malignant tumors constitute an increasing public health problem. In 2022, the number of new cases was 181,300, including 89,794 among men [2]. Prostate cancer was the most frequently registered cancer among men, accounting for 23.3%.

Due to biological and clinical diversity, the main challenge of modern medicine is the ability to make an accurate prognosis [3]. Research to understand the etiology and biology of this cancer is needed to develop preventive methods and treatment strategies.

The etiology of prostate cancer is multifactorial and not fully explained. Some researchers emphasize the role of chronic inflammation, although opinions differ here [4]. Supporters of this theory believe that inflammation plays a key role in the development of prostate cancer [5,6]. Research by Gurel et al. [7] showed that chronic prostatitis increases the risk of PCa by 30%. Prostatitis was classified as acute or chronic, both bacterial and nonbacterial [4].

Disturbances in epithelial cells, changes in their phenotype, gene mutations, as well as external factors may result in dysregulation of the prostate environment, causing inflammation [8,9].

Prostate inflammation can be caused by various factors, including microorganisms such as bacteria and viruses. Among them, sexually transmitted infections (STIs) have been investigated as an etiological factor. A meta-analysis by Caini et al. [10] showed that the risk of prostate cancer was 49% higher in men who had had any sexually transmitted disease.

It is estimated that viral infections play a role in approximately 20–25% of all human cancer cases [11,12]. Oncogenic viruses constitute a taxonomically diverse group with different mechanisms of cancer development [13,14]. Recently, publications have appeared in the medical literature regarding the relationship between prostate cancer and viral infections such as human papillomavirus, cytomegalovirus, human herpes simplex virus type 2, human herpes virus type 8, Epstein–Barr virus, and polyomaviruses [15].

Polyomavirus (PyV) was discovered accidentally in 1950 during research on the infectious agent causing rodent cancer [16]. The *Polyomaviridae* family includes small, non-enveloped viruses. They have a circular dsDNA genome consisting of a non-coding control region (NCCR) and two coding regions—early (T-Ag, t-Ag) and late coding—for three structural proteins (VP1, VP2, and VP3). Large T antigen (T-Ag) is a key protein of all polyomaviruses, essential for viral DNA replication and transcription. Well-studied human polyomaviruses are BK PyV and JC PyV [17].

JCPyV is ubiquitous and it is estimated that 70–80% of the adult population is infected [18]. Primary infection usually appears in childhood and is asymptomatic or mild. The virus can be detected in various organs such as the gastrointestinal tract, spleen, lymph nodes, lungs, bone marrow, brain, B lymphocytes, and kidneys [19].

JCV, BKV, and EBV, are being investigated as potential factors playing a role in the PCa [20,21,22,23,24]. In vitro and in vivo studies have shown that JCPyV has the ability to transform cells [25]. The main oncogenic protein of the virus is large T antigen (T-Ag), which binds retinoblastoma proteins (pRb) and p53, leading to cell cycle dysregulation. Early proteins, small tumor antigen (t-Ag), and agnoprotein are also believed to participate in the cellular transformation process.

In our previous studies, we detected EBV DNA in the tumor tissue in 49% of patients with prostate cancer, which may suggest a role of EBV in the progression of this cancer [26]. Therefore, we decided to examine prostate tumor tissue for the presence of two polyomaviruses, i.e., BKV and JCV. Our goal was also to assess possible co-infection with the studied viruses. Moreover, we compared EBV single-infected and EBV/JCV co-infected patients in the context of selected clinicopathological parameters, i.e., risk group, Gleason score, and TNM classification. We also compared the serological status of patients. We wanted to examine the frequency and level of anti-EBV antibodies in the serum of patients with EBV mono-infection compared to patients with EBV/JCV co-infection. We then analyzed whether co-infection had an effect on anti-EBV antibody levels as well as on EBV load.

## 2. Materials and Methods

### 2.1. Characteristics of Patients

The study included 115 men who were diagnosed and histopathologically confirmed with PCa (adenocarcinoma) by an experienced pathologist. All patients were hospitalized at the Department of General and Oncological Urology of the 1st Military Clinical Hospital with Outpatient Clinic in Lublin from January to November 2023. Patients who had previously undergone chemotherapy or radiotherapy were excluded from the study. All patients underwent radical prostatectomy. The mean age of the patients was 68.9 ± 7.4 years (Table 1).

According to the classification of the European Association of Urology, patients were divided into three risk groups: low-risk group, medium-risk group and high-risk group. This classification is based on the following parameters: prostate-specific antigen (PSA) level, Gleason score (GS), and the TNM staging system [27,28,29]. This categorization system thus distinguishes the following risk groups: the low-risk group—PSA < 10 ng/mL, GS < 7 (ISUP grade 1), and cT1-2a; the intermediate risk group—PSA 10–20 ng/mL, GS 7 (ISUP grade 2/3), or cT2b; the high-risk group—PSA > 20 ng/mL, GS > 7 (ISUP grade 4/5), or cT2c [27,28,29].

PCa patients were assigned to these groups according to viral infection. Subsequent detailed comparison of the two patient groups revealed no significant differences in their sociodemographic characteristics, indicating that the two groups were homogeneous in this respect.

### 2.2. Sample Collection

Clinical materials were collected from patients suffering from PCa. This included serum, as well as fresh frozen tumor tissues. The samples were coded using a sample identification system in order to ensure the anonymity of the patients. The materials were collected and delivered to the laboratory within 24 h. The tissues were collected during surgical procedures, while the blood was collected in accordance with standard hospital procedures during routine examinations. The remaining samples were submitted to our laboratory for further analysis. Centrifugation of the blood samples (1500× *g*/15 min) at room temperature was performed to separate the serum. The clinical material was stored at −80 °C until analysis.

### 2.3. Tissue Selection

Patients were qualified for the study based on the histopathological examination of preoperative biopsy, PSA, and multiparametric MRI (mpMRI imaging technology—currently the most accurate reflection of the structure of the prostate gland) with PI-RADS assessment. Patients with a PI-RADS score of at least 4 (high probability of a clinically significant form of cancer) were qualified for the study.

For the evaluation of the clinical samples, tissue was collected from the prostate gland after the radical prostatectomy with da Vinci robot. The biopsy material is too poor in volume.

The material for our study was collected from the part of the prostate gland in which areas of prostate cancer were macroscopically identified based on its consistency, extent, and correlation with the mpMRI image and then confirmed by histopathological diagnosis. No prostate cancer cells were detected in samples classified as margins.

Only prostate cancer samples that were clearly identified as PCa tissue, confirmed histopathologically, and classified according to EAU guidelines were included in further analysis. Tissue samples meeting the above-mentioned criteria were collected into Eppendorf tubes and frozen at −80 °C until analysis.

### 2.4. Isolation and Detection of EBV DNA

Tumor tissues were cut and homogenized using an Omni TH homogenizer (Omni International, Kennesaw, GA, USA). Subsequently, the QIAamp DNA Mini Kit (Qiagen, Hilden, Germany) was employed for the extraction of DNA, with this process being conducted in accordance with the manufacturer’s protocol. The quality of the extracted DNA was then assessed through the implementation of a β-globin assay, which was utilized to both ensure the integrity of the DNA and to identify the presence of any potential PCR inhibitors. Purified DNA was quantified using Epoch Microplate Spectrophotometer (BioTek Instruments Inc., Vinooski, VT, USA). The extracted DNA was then amplified using the GeneProof Epstein–Barr virus (EBV) PCR Kit (GeneProof, Brno, Czech Republic), which calculates the EBV DNA copy number using the ISEX version of the EBV PCR kit. All samples, including the negative control, were analyzed in duplicate. Amplification of the specific DNA sequence for EBNA1 was performed using LightCycler 2.0 software version 4.1 (Roche Applied Science System, Penzberg, Germany) during the PCR process.

### 2.5. Isolation and Detection BKV and JCV

The preparation of tissues for the detection of JCV and BKV was conducted in a manner consistent with the protocol established for the detection of EBV. The extraction of genetic material was undertaken using the QIAamp DNA Mini Kit (Qiagen, Hilden, Germany), in accordance with the manufacturer’s guidelines. Following this process, the material underwent amplification via the use of the GeneProof BK/JC Virus (BK/JC) PCR Kit (GeneProof, Brno, Czech Republic) in conjunction with the CFX96 Dx ORM (Bio-Rad, Pleasanton, CA, USA).

### 2.6. Detection of Anti-EBV Antibodies

Anti-EBV antibodies were detected using a commercially available Microblot-Array test (TestLine Clinical Diagnostics Ltd., Brno, Czech Republic) according to the manufacturer’s protocol. This test contains a combination of selected parts of the specific antigens of EBV (EBNA-1, EBNA-2, VCA p18, VCA p23, EA-D p54, EA-D p138, EA-R, Rta, ZEBRA, gp85, gp350 and LMP1). This is a quantitative test, and the results are expressed in U/mL. Results below 185 U/mL are considered negative, while results above 210 U/mL are considered positive. Analysis was conducted using the Microblot-Array reader with software version 2.0.4.

### 2.7. Statistical Analysis

GraphPad Prism version 10.4.1 (San Diego, CA, USA) was used for statistical evaluation of the obtained results and graphical presentation of the data. The Shapiro–Wilk test was used to test for a normal distribution of continuous variables. Categorical data were presented as numbers and percentages. The relationship between clinical and demographical parameters was calculated using Pearson’s chi-square test and Fisher’s exact test. All analyzed parameters were presented as arithmetic means, standard deviations (SD), as well as median, lowest, and highest values. To compare differences between the studied groups, the Mann–Whitney U test and/or Kruskal–Wallis test were used. The results were assessed as statistically significant at *p* ≤ 0.05.

### 2.8. Ethics

The study was conducted according to the guidelines of the Declaration of Helsinki and approved by the Medical University of Lublin Ethics Committee (No. KE-0254/194/10/2022, 6 October 2022). Informed consent was obtained from all patients in written form.

## 3. Results

### 3.1. Evaluation of the Frequency EBV, JCV, and BKV in the Tumor Tissue of PCa Patients

**Figure 1:**
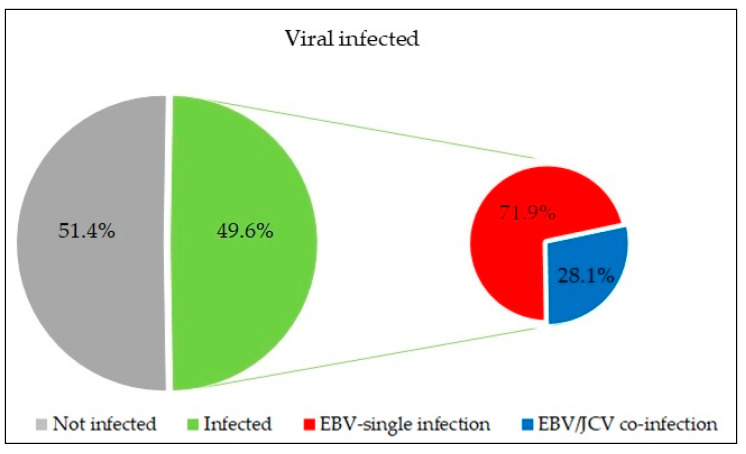
Frequency of EBV single infection and EBV/JCV co-infection in tumor tissue isolated from patients with prostate cancer. Green color—infected patients; gray color—not infected patients; red color—EBV single infection; blue color—EBV/JCV co-infection.

The first goal of our research was to assess the frequency of the genetic material of EB, BK, and JC viruses. Among the 115 patients included in the study, viral DNA was detected in 57 people (49.6%), including 41 (71.9%) with single EBV infection and 16 (28.1%) with EBV/JCV co-infection (Figure 1). However, neither BK virus nor single JCV infections were detected in any of the samples tested.

### 3.2. Comparison of Patients with Single EBV Infection and Patients with EBV/JCV Co-Infection in the Context of Risk Group, Gleason Score, and TNM Classification

Two groups of patients were included for further analysis, i.e., 41 patients with single EBV infection and 16 patients with EBV/JCV co-infection.

Due to the small numbers in the subgroups, two risk groups were created, i.e., the first low-risk group and the second medium-/high-risk group (Table 2). The Gleason score was analyzed similarly, i.e., GS 6—clinically insignificant prostate cancer and GS 7–9—clinically significant prostate cancer. The allocation of patients diagnosed with PCa to these groups was made according to the above-mentioned classification system.

In the studied patient groups, there were no T3 or T4 stages, no metastases to regional lymph nodes (N0 = 100%), and no distant metastases (M0 = 100%).

The objective of the present analysis was to ascertain whether EBV/JCV co-infection has an impact on the clinical features presented in Table 2. The present study revealed differences in pathological features. In the group of patients with single EBV infection, 75.6% of patients were classified as intermediate/high risk, while 56.2% of patients with EBV/JCV co-infection were classified as low risk (*p* = 0.0307).

Moreover, 58.5% of patients with single EBV infection were classified into group GS 7–9, while 75.0% of patients with EBV/JCV co-infection were designated as being in the GS 6 group. The data indicate a significant difference in the Gleason score between the group of patients with single EBV infection and co-infection. Among patients with single EBV infection, a more advanced stage of cancer predominated (*p* = 0.0379).

A similar tendency was noticed regarding tumor size (T). The T2 group constituted 73.2% of patients infected only with EBV, while patients with EBV/JCV co-infection constituted 62.5% of the T1 group (*p* =0.0166).

### 3.3. Frequency of Anti-EBV Antibodies in PCa Patients with EBV Single Infection Compared with EBV/JCV Co-Infection

In the next stage of our study, we wanted to check the frequency and the level of anti-EBV antibodies in patients with single EBV infection compared to patients with EBV/JCV co-infection in the context of risk group, Gleason Score, and tumor size (T). First, we assessed the prevalence of anti-EBV antibodies.

**Figure 2:**
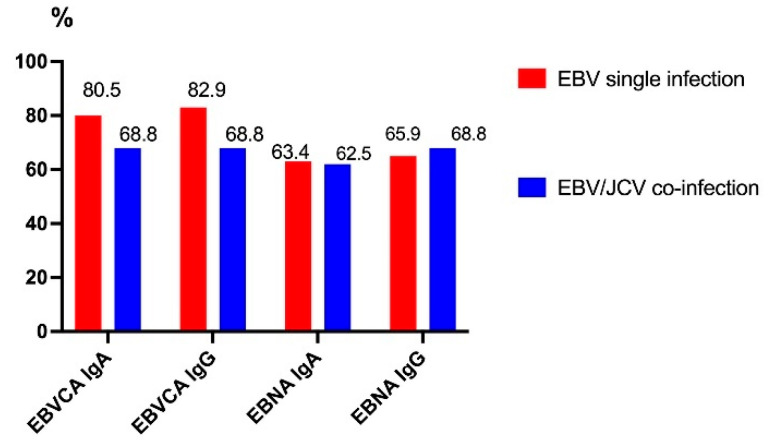
Prevalence of EBVCA and EBNA1 antibodies in PCa patients. Red color—EBV single infection; blue color—EBV/JCV co-infection.

Among the numerous antigens contained in the diagnostic kit used, only antibodies against the two major EBV antigens, namely EBVCA and EBNA, were detected in the sera of the patients studied, both in the IgG and IgA classes. The presence of anti-EBV IgM antibodies was not detected in any of the patients studied. The seroprevalence results are presented in Figure 2.

Despite the fact that anti-EBVCA antibodies were detected more often in people with single EBV infection than in co-infection, this difference was not statistically significant. However, the frequency of anti-EBNA antibodies in both groups of patients was similar.

Then, we analyzed the frequency of the studied antibodies in the context of risk groups, GS, and T stages.

Table 3 shows the prevalence of antibodies analyzed by risk group. In both the serum of patients with single EBV infection and co-infection, anti-EBV antibodies were detected more often in high-risk groups. Anti-EBNA antibodies, both IgA (*p* = 0.0036) and IgG (*p* = 0.159), were statistically significantly more common in the intermediate-/high-risk group of patients with single EBV infection.

Then, we assessed the prevalence of anti-EBV antibodies in relation to Gleason score (Table 4). It was observed that, in patients with a single EBV infection, anti-EBVCA IG and anti-EBNA IgA antibodies were more frequently detected at a more advanced stage (GS 7–9). In the case of EBV/JCV co-infection, such a relationship did not occur.

As illustrated in Table 5, a similar tendency was observed regarding the T trait. More often, the analyzed antibodies occurred at a more advanced stage (T2). This difference is particularly evident with respect to IgA and IgG EBNA in a single EBV infection. In the group of patients with EBV/JCV co-infection, anti-EBVCA IgG antibodies were detected in all people with the T2 feature (*p* = 0.0367).

### 3.4. Antibody Levels for EBVCA IgA and IgG as Well as EBNA1 IgA and IgG in PCa Patients

The next stage of our research concerned the level of anti-EBVCA and anti-EBNA antibodies (both IgA and IgG) in single EBV infection and EBV/JCV co-infection in relation to selected clinicopathological features such as risk group, Gleason score, and T stage.

The mean level of EBVCA IgA was found to be 760.08 U/mL in the EBV single infection group and 624.85 U/mL in the EBV/JCV co-infection group. Conversely, the mean average EBVCA IgG level in the EBV-positive group was found to be 760.79 U/mL, whereas in the EBV/JCV co-infection group, this level was determined to be 611.81 U/mL. Furthermore, the mean EBNA IgA level was 675.92 U/mL (EBV-positive group) and 471.26 U/mL (EBV/JCV co-infection group). In contrast, the mean average EBNA IgG level in the EBV single infection group was 659.70 U/mL, and 518.96 U/mL in the EBV/JCV co-infection group.

**Figure 3:**
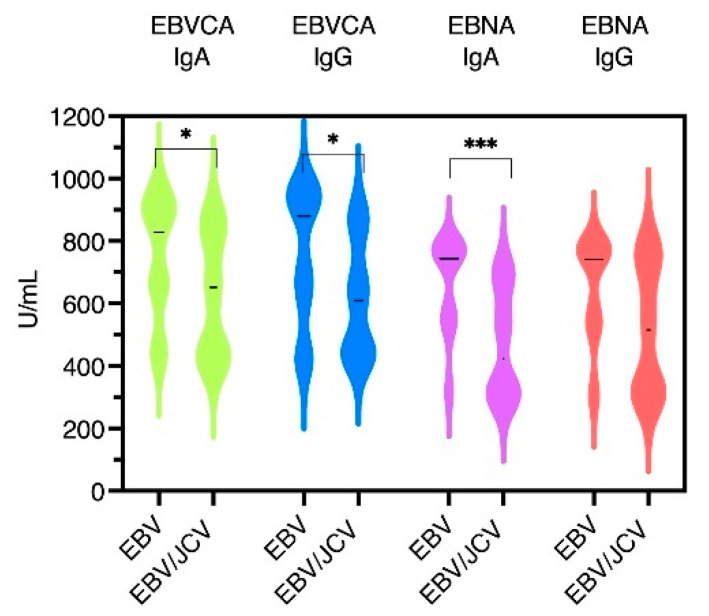
Levels of EBVCA and EBNA1 antibodies in PCa patients with single EBV infection compared to EBV/JCV co-infected patients; * *p* = 0.01, *** *p* = 0.001.

Thus, statistical analysis of the antibody levels showed a statistically significant difference between these antibodies (Figure 3).

**Figure 4:**
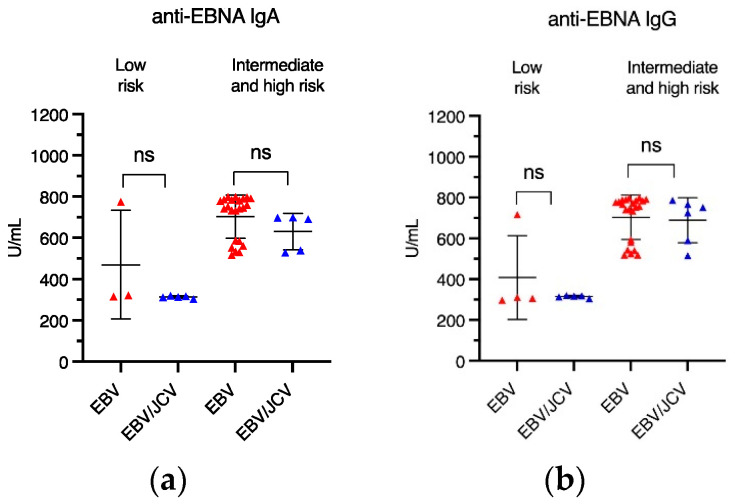

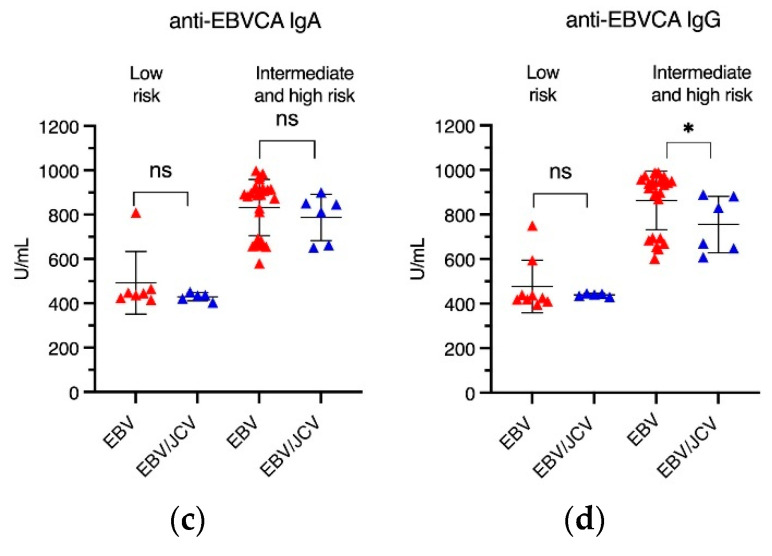
The level of antibodies in relation to the risk group: (**a**) EBNA1 IgA, (**b**) EBNA1 IgG, (**c**) EBVCA IgA, and (**d**) EBVCA IgG; Mann–Whitney U test. Red color—EBV single infection; blue color—EBV/JCV co-infection, * *p* = 0.01, ns—not statistically significant.

Next, we assessed whether there were differences in anti-EBV levels among risk groups (Figure 4). Statistically significant differences were observed only in the level of anti-EBVCA IgG antibodies in the intermediate-/high-risk group; the titer of these antibodies was higher in a single EBV infection (Figure 4d). Further details on antibody titers can be found in Table S1 in the Supplementary Material.

**Figure 5:**
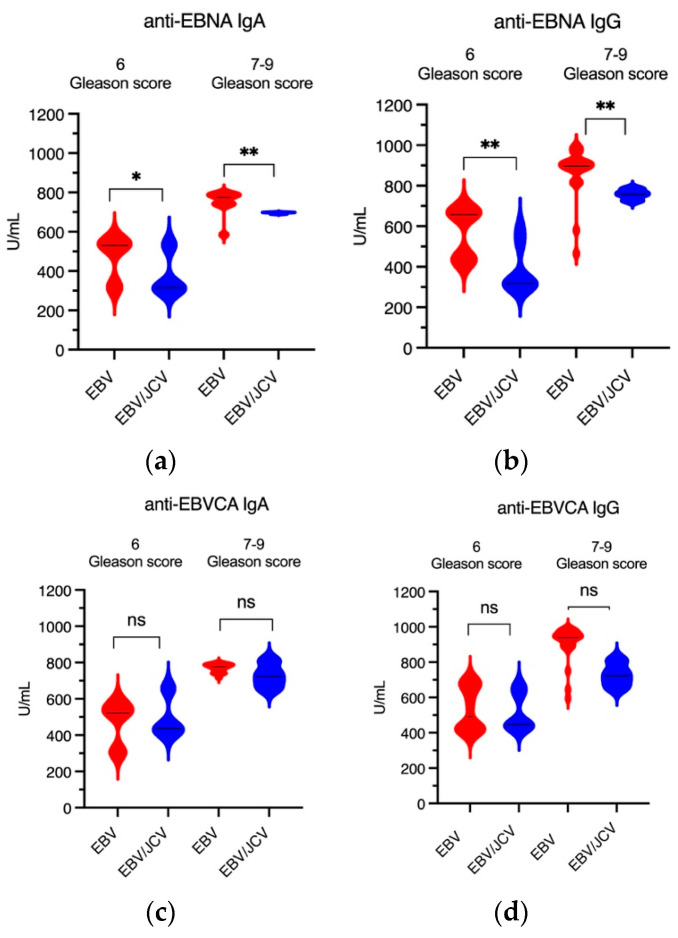
The level of anti-EBV antibodies in relation to GS: (**a**) EBNA1 IgA, (**b**) EBNA1 IgG, (**c**) EBVCA IgA, and (**d**) EBVCA IgG; Mann–Whitney U test. Red color—EBV single infection; blue color—EBV/JCV co-infection; * *p* = 0.01, ** *p* = 0.001, ns—not statistically significant.

Figure 5 shows the antibody level in PCa patients with EBV single infection and EBV/JCV co-infection depending on the Gleason score. It was observed that in patients with single EBV infection, both the concentration of anti-EBNA and EBVCA antibodies (in both classes) was significantly higher than in EBV/JCV co-infection. It should be added that a higher level of these antibodies occurred at a more advanced stage, i.e., GS 7–9 (clinically significant prostate cancer) (Figure 5a,b,d). A detailed analysis of the data can be found in Supplementary Table S2.

**Figure 6:**
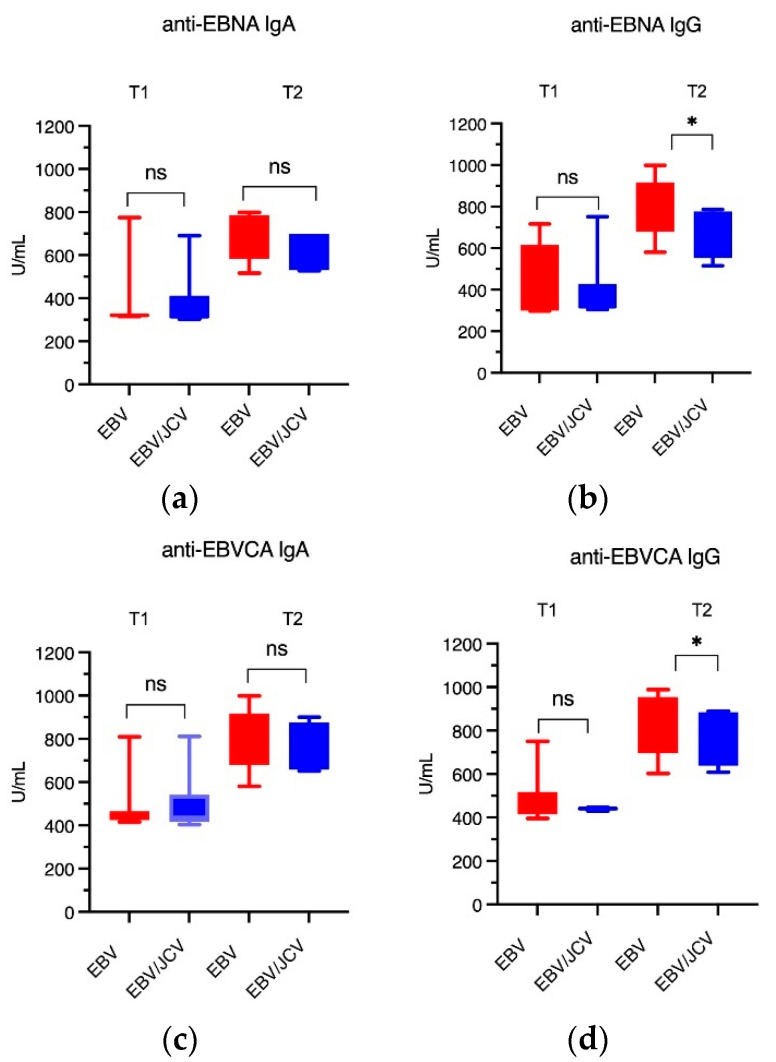
The level of anti-EBV antibodies in the relation to the T stage: (**a**) EBNA1 IgA, (**b**) EBNA1 IgG, (**c**) EBVCA IgA, (**d**) EBVCA IgG; Mann–Whitney U test. Red color—EBV single infection; blue color—EBV/JCV co-infection, * *p* = 0.01; ns—not statistically significant.

In turn, Figure 6 shows the antibody titer in relation to the T trait. The highest antibody levels were observed in patients with EBV mono-infection. Specifically, the titers of EBNA IgG and EBVCA IgG were significantly higher at stage T2 in single EBV infection (Figure 6b,d). A detailed analysis of the data can be found in Supplementary Table S3.

### 3.5. Comparison of EBV Load in Tumor Tissue in PCa Patients with Single EBV Infection and in Patients with EBV/JCV Co-Infection

**Figure 7:**
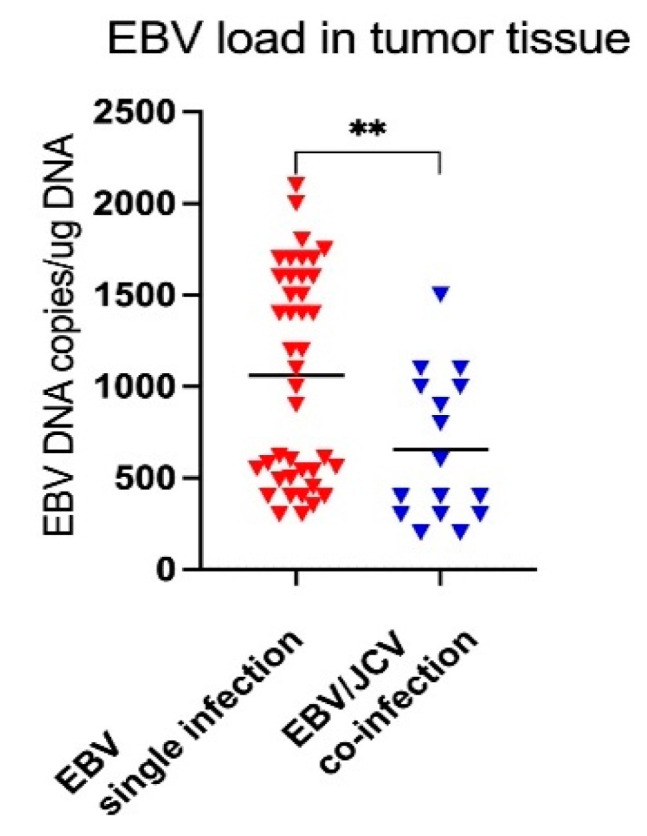
Tumor tissue EBV load in single EBV infection and in EBV/JCV co-infection. ** *p* = 0.0068; Mann–Whitney U test.

At the final point of our study, we assessed EBV load in the collected tumor tissue. EBV load was compared in tissue samples in which only EBV was detected and in samples with EBV/JCV co-infection. The obtained results of this analysis are presented in Figure 7. The result is shown as EBV DNA copies per 1 µg of DNA in tumor tissue. The analysis of the obtained results shows that in malignant tissues in which only EBV DNA was detected, its concentration was statistically significantly higher than in cancer tissues co-infected with EBV/JCV. The average EBV viral load in single EBV infection was 1060 copies EBV DNA/ug tumor tissue and 656 copies EBV DNA/ug tissue in EBV/JCV co-infection.

## 4. Discussion

There are many reports in the available scientific literature on the detection of oncogenic viruses in prostate cancer [30]. Many researchers point to the potential role of the JCV virus in causing prostatitis, which may consequently lead to the development of prostate cancer. Despite extensive research on polyomaviruses, their role in the pathogenesis of cancer has not yet been fully elucidated [31].

John Cunningham virus (JCPyV) and BKPyV have been described in 1971 [32,33]. JCV, the first human polyomavirus, was isolated from the brain tissue of a patient with Hodgkin’s disease, and BK virus was isolated from the urine of a kidney transplant patient.

To assess the carcinogenic risk of BKV and JCV infection to humans, the IARC Monograph Working Group [34] analyzed epidemiological evidence and animal bioassays. Both viruses mentioned above have been classified as group 2B, as potentially carcinogenic to humans. The possible association of BK and JC viruses with human cancer is difficult to establish due to the common presence of these viruses in the healthy population [35,36]. Both BKV and JCV infections are usually asymptomatic, although virus particles may be excreted in the urine. In immunocompromised people, latent polyomavirus infection may be reactivated, which may result in high viral load and viruria.

Already in 2015, Anzivino et al. [37] demonstrated the presence of JCV DNA in samples obtained from PCa tissue. These and subsequent studies initiated a discussion on the possible role of JCV in the pathogenesis of P risk groups Ca.

The results regarding the incidence of JCV in PCa are inconsistent between studies. This variability can be attributed to a variety of factors, including differences in sample size and different detection methods. In a study conducted by Delbue et al. [19], JCV was detected in 16.4% of prostate tissue samples tested. However, the study by Zambrano et al. [20] showed a much higher incidence of JCV in PCa at 50%.

In our own research presented here, we detected the presence of viral DNA in 49.6% of clinical samples from freshly frozen prostate cancer tissue, including 71.9% with single EBV infection and 28.1% with EBV/JCV co-infection. We did not detect BKV DNA or a single JCV infection. Samples were taken from tumor areas. Normal tissue adjacent to the tumor (margin) collected from 70 patients served as internal control. In this location, EBV DNA was detected only in nine cases, i.e., at 12.8%. However, no JCV DNA was detected. But, as studies by Shen et al. [38] have shown, PCa cells are more susceptible to JCPyV infection than benign tissues.

Therefore, having detected EBV/JCV co-infection, we compared patients with EBV mono-infection with EBV/JCV co-infected patients in the context of selected clinical features, i.e., risk group, Gleason score, and TNM classification.

Our results showed differences in clinicopathological features between single EBV infection and EBV/JCV co-infection. In the EBV single infection group, most patients were classified as intermediate/high risk, while in the EBV/JCV co-infection group, low-risk patients predominated.

An important indicator for assessing the prognosis of PCa is the Gleason score; a higher score indicates a worse prognosis. In our study, GS 7–9 (clinically significant prostate cancer) was diagnosed in 58.5% of patients with EBV mono-infection. However, in EBV/JCV co-infection, GS 6, i.e., clinically insignificant, was diagnosed in 75% of men.

Other researchers obtained different results. Gorich et al. [21] observed a higher level of LTag JCV with higher viral load in a group of PCa patients compared to the control group. However, viral load did not depend on the Gleason score, which may indicate that JCV infection does not influence tumor progression. In contrast, the study by Shen et al. [38] showed correlation between the presence of JCPyV DNA in PCa tissue and the progression and prognosis of prostate cancer.

In our research, among patients with single EBV infection, a more advanced stage of cancer was observed than in patients with EBV/JCV co-infection. Moreover, as the analysis showed, the level of anti-EBVCA and anti-EBNA antibodies was higher in single infection compared to EBV/JCV co-infection. This tendency also occurred in relation to Gleason score and T stage. Higher antibody levels were detected in more advanced tumor stages in single EBV infection. In addition, higher EBV load was observed in single EBV infection.

Many studies indicate that the detection of anti-EBV antibodies in patients’ serum is not only a useful marker in early diagnosis, but also in monitoring the recurrence and progression of EBV-related cancers [39]. Therefore, we wanted to check what the serological status of PCa patients is and whether it depends on selected clinical features in EBV mono-infection compared to EBV/JCV co-infection.

Our previous study showed both a higher incidence and higher antibody titer in EBV-positive PCa patients compared to the EBV-negative group [26]. Therefore, we wanted to test whether JCV co-infection affects the humoral response against EBV. Even though the current study included a different group of patients, we observed the same tendency. Moreover, the antibody titer depended on pathological features, such as Gleason score, risk groups, and T stage. Interestingly, we did not detect antibodies indicating reactivation of EBV infection, i.e., EA, Zta, and Rta. Perhaps only latent infection plays a role in PCa.

Although anti-EBVCA and anti-EBNA antibodies are of epidemiological importance, it is worth emphasizing that EBNA1 plays a dual role. On the one hand, it maintains latency, and on the other hand, it plays a role in virus reactivation and lytic infection [40,41]. EBNA1, responsible for viral replication, is the only protein expressed in all EBV-positive tumors and is sometimes the only protein expressed at all.

Persistent infections, of which both EBV and JCV are capable, can significantly impact tumor initiation and progression. Molecular studies of various types of cancer have shown that the process of carcinogenesis is influenced by genetic and epigenetic changes in cancer cells, as well as rearrangement of components of the tumor microenvironment (TME) [42]. The TME consists of a cellular part containing tumor cells and stromal cells embedded in the extracellular matrix—a non-cellular component. The interactions between all of these elements rely on a complex network of cytokines, growth factors, inflammatory mediators, and matrix remodeling enzymes and can promote the development and invasion of cancer cells. EBV latent genes and viral miRs expression target multiple intracellular pathways in EBV-infected cells, creating an immunosuppressive environment conducive to the development of EBV-related malignancies [43,44,45]. In the TME, a constantly modified, complex ecosystem, various molecules interact with each other to promote tumor growth and progression. There is growing evidence to suggest that infiltrating immune cells in the prostate parenchyma contribute to disease progression and treatment resistance [46]. Given the known tropism of the Epstein–Barr virus for immune cells (latent infection in B lymphocytes), this may provide a biological justification for our observations. It therefore appears that EBV may play a role in the progression of this disease.

On the other hand, the interaction of host cells and viral factors creates an environment favorable to tumorigenesis. It can be assumed that JCV also interacts in a similar way. Viral products, such as large T antigen of polyomaviruses, can transform prostate cells and interfere with the interferon (IFN) [47]. Numerous studies have shown that cell transformation is the result of JCPyV-induced genome instability [48]. Large T antigen (T-Ag) binds to β-catenin, induces its translocation into the host cell nucleus, which in turn enhances the c-MYC gene, which, as a strong proto-oncogene, participates in cell cycle control, DNA metabolism, and apoptosis [49,50,51,52].

The virus–virus relationship may involve direct interactions of genes or their products, changes in the host environment and/or tumor microenvironment, and immunological interactions [53,54].

When a cell is infected with two different viruses, interference can occur in which one virus inhibits the replication of the other virus. The phenomenon of interference between two viruses may occur at any stage of the virus replication cycle. Viral interference also often occurs in persistent infections, where viruses remain in infected cells in a state of latency, reducing the level of replication to keep the infected cell alive. Unlike interference, co-infection with some viruses can enhance the replication of other viruses. The best examples are respiratory viruses [55]. Another well-known and documented phenomenon is co-infection or super-infection with HBV/HCV [56].

In the present case, when the prostate cells are infected with EBV and JCV, interference may also occur, especially since both EBV and JCV have the ability to establish a state of latency in the host cell.

Due to the fact that human polyomavirus DNA has been found in many different cell types, co-infection with other oncogenic viruses is possible [57]. Therefore, it is assumed that polyomaviruses may act as a co-factor in the development of cancer, including cancer caused by other oncogenic viruses.

Although many researchers have shown a high prevalence of EBV in prostate cancer, its role is also controversial [24,58,59]. Among the examined patients, EBV DNA was detected in 49.5% of cases. However, a co-infection of EBV/JCV was observed in 13.9% of all PCa cases, but no single JCV infection was detected. This fact may suggest that, although JCV co-occurs with other viral infections, it does not necessarily appear to be the sole or main viral factor in the development of PCa. It can be assumed that JCV inhibits EBV replication. This could be evidenced by the lower level of anti-EBV antibodies in EBV/JCV co-infection, especially anti-EBNA. This raises many questions. Does JCV only “reside” in prostate cells or is it a co-factor in EBV infection? Was EBV infection primary and JCV secondary or vice versa? In light of these studies, there is a need to clarify the unclear role of the JCV virus in the development and/or progression of prostate cancer.

According to Pietropaolo et al. [60], immunosuppression resulting from the underlying disease or treatment plays a key role in the reactivation of the latent phase of JCV infection. Unfortunately, how JCV latency is reactivated is not well understood.

There is no direct cause-and-effect relationship between infection with any virus and the development of cancer. It is, rather, persistent viral infections that promote cancer. Cancer transformation is a complex and multi-stage process in which the virus may be one of the factors. Neither EBV nor poliomavirus play a direct oncogenic role in prostate cancer.

To properly determine whether the JCV virus is indeed a risk factor for prostate cancer or is involved in co-infection, the following issues should be taken into account. It is necessary to assess whether PCa patients were exposed to contact with JCV, which will be indirectly confirmed by the presence of specific antibodies. Strategies to prove or exclude “hit-and-run” oncogenesis seem to be an interesting challenge [61]. The “hit and run” theory suggests that viruses encourage the accumulation of mutations, which causes genome instability until the virus is no longer needed for the cell to survive. Currently, there is insufficient evidence to determine whether these viruses act as “drivers” or “passengers” in the process of prostate carcinogenesis. However, despite many unresolved issues, it seems that the role of polyomaviruses in the etiopathogenesis of prostate cancer, although unlikely, is not impossible.

### 4.1. Limitation of Our Study

These studies were conducted only in one clinical center, which does not allow generalization of the conclusions. The study group was relatively small, especially the one in which EBV/JCV co-infection was detected. JCV load was not analyzed quantitatively, only qualitatively. In tissue from PCa patients, JCV viral load was classified as low or high based on the cycle threshold (Ct) value of the viral gene. There were only 16 cases of co-infection and low JCV viremia was found in all samples. The research presented here does not include tissue samples from benign prostatic hyperplasia (BPH), whereas the control group (internal control) was a margin of healthy tissue, as described in the Discussion.

Given both the results obtained and the limitations of this study, this will require further research on a much larger group of patients. Only further detailed, in-depth studies can clarify the clinical significance of JCV in prostate cancer.

### 4.2. Future Research Directions

This manuscript presents preliminary results of research that will be continued. Despite many studies, the hypothesis that polyomaviruses, especially BKV and JCV, may contribute to the development of prostate cancer is controversial. Further research is needed to evaluate the complex interplay between host genetics, host immunity, and viral infection that may lead to the initiation or promotion of prostate cancer.

Future studies will include assessment of viral gene expression, virus presence in tumor cells (e.g., using ISH), and JCV load. A larger number of patients will enable the assessment of the correlation between EBV load and JCV load. Clinical material is already being collected for this purpose. Moreover, future studies should increase the number of clinical parameters analyzed to assess prognosis in patients with EBV mono-infection and EBV/JCV co-infection.

## 5. Conclusions

The role of JCV in the development of PCa remains unclear due to the paucity of research in this area and divergent results among individual researchers. Some studies have shown a potential correlation between the JCV virus and the development and/or progression of prostate cancer. The results of our study indicate that EBV/JCV co-infection probably does not have a significant impact on the development of PCa. On the other hand, our studies may indirectly confirm the involvement of the EBV virus in the progression of prostate cancer. Lower levels of anti-EBV antibodies and lower EBV viral load in co-infection may suggest that JCV inhibits EBV replication. However, these observations require confirmation in a larger group of cases. Further studies can elucidate the clinical significance of JCV and its possible role in PCa as well as in co-infection with other viruses.

## Figures and Tables

**Table 1:** Epidemiological characteristics of PCa patients.

		PCa Patients			
		EBV Single Infection		EBV/JCV Co-Infection	
		*n*	%	*n*	%
Total		41	35.7	16	13.9
Age	<60	6	14.6	1	6.3
	>60	35	85.4	15	93.8
*p*		0.6599			
Place of residence	Urban	28	68.3	9	56.3
	Rural	13	31.7	7	43.8
*p*		0.5379			
Smoking	Never	10	24.4	2	12.5
	Ever	31	75.6	14	87.5
*p*		0.4767			
Alcohol abuse	never	14	34.2	5	31.3
	≤drink per week	23	56.1	10	62.5
	>drink per week	4	9.8	1	6.3
*p*		0.9999			

Pearson’s chi-squared test.

**Table 2:** Clinical characteristics of PCa patients with EBV single infection and EBV/JCV co-infection.

		PCa Patients			
		EBV Single Infection N = 41		EBV/JCV Co-Infection N = 16	
		*n*	%	*n*	%
Risk	Low	10	24.4	9	56.2
	Intermediate/high	31	75.6	7	43.8
*p*		0.0307 *			
Gleason score	6	17	41.5	12	75.0
	7–9	24	58.5	4	25.0
*p*		0.0379 *			
T	T1	11	26.8	10	62.5
	T2	30	73.2	6	37.5
	T3	0	0.0	0	0.0
	T4	0	0.0	0	0.0
*p*		0.0166 *			
N	N0	41	100.0	16	100.0
M	M0	41	100.0	16	100.0

* Statistically significant; Pearson’s chi-squared test.

**Table 3:** Prevalence of EBVCA and EBNA1 antibodies in PCa patients according to the risk group.

	EBV-Positive					EBV/JCV Co-Infection				
	Low Risk		Intermediate/ High Risk		*p*	Low Risk		Intermediate/ High Risk		*p*
	*n*	(%)	*n*	(%)		*n*	(%)	*n*	(%)	
	*n* = 10		*n* = 30			*n* = 9		*n* = 7		
EBVCA IgA	7	70.00	26	86.67	0.2297	5	55.56	6	85.71	0.1967
EBVCA IgG	9	90.00	25	83.33	0.9090	5	55.56	6	85.71	0.1967
EBNA IgA	3	30.00	23	83.33	0.0036 *	5	55.56	5	71.43	0.5153
EBNA IgG	4	40.00	23	83.33	0.0159 *	5	55.56	6	85.71	0.1967

* Statistically significant; Pearson’s chi-squared test.

**Table 4:** Prevalence of EBVCA and EBNA1 antibodies in PCa patients according to GS.

	EBV Single Infection					EBV/JCV Co-Infection				
	6 Gleason Score		7–9 Gleason Score		*p*	6 Gleason Score		7–9 Gleason Score		*p*
	*n*	(%)	*n*	(%)		*n*	(%)	*n*	(%)	
	*n* = 18		*n* = 23			*n* = 12		*n* = 4		
EBVCA IgA	12	66.67	13	56.52	0.5087	7	58.33	4	100.00	0.1195
EBVCA IgG	12	66.67	22	95.65	0.0313 *	8	66.67	3	75.00	0.7555
EBNA IgA	8	44.40	18	78.26	0.0486 *	7	58.33	3	75.00	0.5510
EBNA IgG	10	55.56	17	73.91	0.2186	7	58.33	4	100.00	0.1195

* Statistically significant; Pearson’s chi-squared test.

**Table 5:** Prevalence of EBVCA and EBNA1 antibodies in patients with PCa according to T stage.

	EBV Single Infection					EBV/JCV Co-Infection				
	T1		T2		*p*	T1		T2		*p*
	*n*	(%)	*n*	(%)		*n*	(%)	*n*	(%)	
	*n* = 11		*n* = 30			*n* = 10		*n* = 6		
EBVCA IgA	7	63.64	26	86.67	0.0992	6	60.00	5	83.33	0.3296
EBVCA IgG	9	81.82	25	83.33	0.9090	5	50.00	6	100.00	0.0367 *
EBNA IgA	3	27.27	23	76.67	0.0036 *	6	60.00	4	66.67	0.7897
EBNA IgG	4	36.36	23	76.67	0.0159 *	6	60.00	5	83.33	0.3296

* Statistically significant; Pearson’s chi-squared test.

## Data Availability

The data presented in this study are available in the article.

## Supplementary Materials

The following supporting information can be downloaded at: https://www.mdpi.com/article/10.3390/cancers17101725/s1, Table S1: Analysis of EBVCA and EBNA1 antibodies levels in PCa patients with EBV single infection and EBV/JCV co-infection, according to risk groups; Table S2: Analysis of EBVCA and EBNA1 antibodies levels in PCa patients with EBV single infection and EBV/JCV co-infection, according to Gleason score; Table S3: Analysis of EBVCA and EBNA1 antibodies levels in PCa patients with EBV single infection and EBV/JCV co-infection, according to the T stage.
