# Supplementary material for: JC Polyomavirus in Prostate Cancer—Friend or Foe?
Source: Cancers (Basel). 2025 May 21;17(10):1725. doi: 10.3390/cancers17101725 (PMC12109926; doi:10.3390/cancers17101725)
Supplement: Supplementary file 1 [file cancers-17-01725-s001.zip › cancers-3659546-supplementary.pdf]

Supplementary Materials Table S1: Analysis of EBVCA and EBNA1 antibodies levels in prostate cancer patients with single EBV and EBV/JCV co-infection, according to risk groups.

		EBV single infection							EBV/JCV co-infection						
**Group**	**Parameter**	**n**	**Mean**	**S D**	**Median**	**Min**	**Max**	**p value**	**n**	**Mean**	**S D**	**Median**	**Min**	**Max**	**p value**
**EBVCA IgA**	**Low risk**	7	492,5	140,8	445,2	415,6	809,6	<0,0001*	5	429,9	18,2	435,3	403,7	451,7	0,0043*
	**Intermediate/high risk**	26	832,1	126,5	889,5	580,6	998,8		6	787,3	104,8	829,1	651,9	900,3	
	**p value**	<0,0001*													
**EBVCA IgG**	**Low risk**	9	477,0	118,4	426,9	395,0	750,7	<0,0001*	5	439,9	6,7	441,9	430,3	446,4	0,0043*
	**Intermediate/high risk**	25	862,9	131,3	933,0	602,3	988,1		6	755,1	126,5	750,6	608,8	889,3	
	**p value**	<0,0001*													
**EBNA IgA**	**Low risk**	3	469,9	263,6	320,1	315,2	774,3	0,1131	5	312,1	6,0	312,2	302,7	318,2	0,0079*
	**Intermediate/high risk**	23	702,8	105,3	745,7	516,6	798,2		5	630,4	89,1	690,2	527,7	698,7	
	**p value**	0,0009*													
**EBNA IgG**	**Low risk**	4	408,0	205,7	309,3	297,1	716,4	0,0043*	5	314,8	6,3	317,7	304,5	319,8	0,0043*
	**Intermediate/high risk**	23	703,5	109,2	756,5	518,1	799,6		6	689,1	110,3	738,6	515,4	786,7	
	**p value**	0,0005*													

Supplementary Materials Table S2: Analysis of EBVCA and EBNA1 antibodies levels in PCa patients with EBV single infection and EBV/JCV co-infection, according to Gleason score.

		EBV single infection							EBV/JCV co-infection						
**Group**	**Parameter**	**n**	**Mean**	**S D**	**Median**	**Min**	**Max**	**p value**	**n**	**Mean**	**S D**	**Median**	**Min**	**Max**	**p value**
**EBVCA IgA**	**6 Gleason score**	12	571,0	121,3	657,1	415,6	692,0	<0,0001*	7	494,8	111,9	437,6	403,7	662,1	0,0061*
	**7-9 Gleason score**	21	868,1	126,4	896,3	465,3	998,8		4	852,5	36,4	848,8	812,0	900,3	
	**p value**	<0,0001*													
**EBVCA IgG**	**6 Gleason score**	12	528,9	134,1	438,5	395,0	696,0	<0,0001*	8	515,9	106,3	445,6	430,3	445,6	0,0121*
	**7-9 Gleason score**	22	887,3	122,7	936,5	594,9	988,1		3	867,6	32,2	882,9	106,3	889,3	
	**p value**	<0,0001*													
**EBNA IgA**	**6 Gleason score**	8	497,4	112,8	542,5	315,2	586,1	<0,0001*	7	375,2	375,2	316,5	302,7	538,2	0,0167*
	**7-9 Gleason score**	18	755,3	64,0	776,4	516,6	798,2		3	695,4	4,6	697,4	690,2	698,7	
	**p value**	<0,0001*													
**EBNA IgG**	**6 Gleason score**	10	473,6	118,9	521,7	297,1	593,7	<0,0001*	7	382,6	117,9	318,7	318,7	589,1	0,0061*
	**7-9 Gleason score**	17	769,2	24,7	776,4	716,4	799,6		4	757,5	25,7	758,8	725,7	786,7	
	**p value**	<0,0001*													

Supplementary Materials Table S3: Analysis of EBVCA and EBNA1 antibodies levels in PCa patients with EBV single infection and EBV/JCV co-infection, according to T stage

		EBV single infection							EBV/JCV co-infection						
**Group**	**Parameter**	**n**	**Mean**	**S D**	**Median**	**Min**	**Max**	**p value**	**n**	**Mean**	**S D**	**Median**	**Min**	**Max**	**p value**
**EBVCA IgA**	**T1**	7	492,5	140,8	445,2	415,6	809,6	<0,0001*	6	493,6	156,8	436,4	403,7	812,0	0,0173*
	**T2**	26	832,1	126,5	889,5	580,6	998,8		5	782,4	116,4	846,2	651,9	846,2	
	**p value**							<0,0001*							
**EBVCA IgG**	**T1**	9	477,0	118,4	395,0	395,0	750,7	<0,0001*	5	439,9	6,7	441,9	430,3	446,4	0,0043*
	**T2**	25	862,9	131,3	933,0	602,3	988,1		6	755,1	126,5	750,6	608,8	750,6	
	**p value**							<0,0001*							
**EBNA IgA**	**T1**	3	469,9	263,6	320,1	315,2	774,3	0,1131	6	375,1	154,5	314,3	302,7	690,2	0,0381*
	**T2**	23	702,8	105,3	745,7	263,6	798,2		4	615,5	95,4	617,8	527,7	698,7	
	**p value**							0,0017*							
**EBNA IgG**	**T1**	4	408,0	205,7	309,3	297,1	716,4	0,0043*	6	387,6	178,4	318,2	304,5	751,5	0,0303*
	**T2**	23	703,5	109,2	756,5	518,1	799,6		5	676,6	118,5	725,7	515,4	786,7	
	**p value**							0,0012*							
